# AI-Generated Multiple Mini Interview (MMI) Stations for Medical School Admissions: Psychometric Evaluation

**DOI:** 10.2196/86208

**Published:** 2026-06-11

**Authors:** Sabryn Hamila, Kyle Birchill, Khoa Cao, Md Nassif Hossain, Shane Bullock, Wayne Hodgson, Julia Harrison, Michelle Leech

**Affiliations:** 1 School of Medicine, Faculty of Medicine, Nursing and Health Sciences, Monash University, 27 Rainforest Walk, Clayton, 3800, Australia, 61 404065979; 2 School of Public Health and Preventive Medicine, Faculty of Medicine, Nursing and Health Sciences, Monash University, Clayton, Australia; 3 School of Rural Health, Faculty of Medicine, Nursing and Health Sciences, Monash University, Warragul, Victoria, Australia; 4 Sub-Faculty of Health Sciences, Faculty of Medicine, Nursing and Health Sciences, Monash University, Clayton, Victoria, Australia

**Keywords:** artificial intelligence, AI, medical school admissions, medical education, multiple mini interviews, MMI, assessment reliability, educational technology, generative artificial intelligence, generative AI, psychometric evaluation, medical education innovation

## Abstract

**Background:**

Multiple mini interviews (MMIs) are widely used in medical school admissions to assess applicants’ nonacademic attributes in a structured and reliable manner. However, the development of high-quality MMI stations is resource intensive and dependent on expert input.

**Objective:**

This study explored the utility of artificial intelligence (AI) in the generation of MMI stations for the Direct and Graduate Entry Medicine Program admissions process for domestic applicants at Monash Medical School. To our knowledge, this study represents the first empirical evaluation of AI-generated MMI stations deployed in a real-world medical school admissions context.

**Methods:**

A total of 56 MMI stations from the 2025 admissions cycle were evaluated, including 17 (30.4%) AI-generated and 39 (69.6%) traditionally developed stations, administered across 824 domestic applicants for a total of 4897 applicant-station interactions. We assessed station quality through both reliability (using Cronbach α to examine internal consistency) and discrimination capability (using SD and range of scores) at the station level.

**Results:**

AI-generated stations exhibited slightly higher reliability (α=0.82) compared with traditional stations (α=0.81), though this difference was not statistically significant (*P*=.91). Both AI-generated and traditionally developed stations demonstrated variable discrimination capability, with some stations from each development method showing excellent combinations of high reliability and strong discriminatory power, while others exhibited ceiling effects that limited their discriminatory power. Of note, a greater proportion of AI-generated stations were classified as optimal (α>0.85), and a smaller proportion were classified in the review category (α<0.75), compared with traditional stations. These results suggest that AI-generated stations can achieve psychometric performance comparable to traditionally developed stations.

**Conclusions:**

Our findings highlight the utility of AI as a useful tool for MMI station generation, offering a scalable approach that may reduce the resource burden on faculty while maintaining or enhancing psychometric quality for applicants. Ongoing quality assurance and evaluation remain essential to ensure fairness and validity across the admissions process.

## Introduction

Medical school admissions are evolving to meet the demands of a changing health care landscape. With increasing competition and a growing emphasis on nonacademic traits that predict professional competence and patient care quality, new tools are reshaping how candidates are selected. The multiple mini interview (MMI) format has been widely adopted in medical school admissions and can assess a range of nonacademic skills, such as communication, ethical reasoning, teamwork, and empathy [1-5]. MMI stations typically consist of a stem or prompt that outlines the scenario, followed by several structured questions that candidates must respond to in real time and that are designed to probe different aspects of the candidate’s reasoning or behavior, testing their abilities to think critically, demonstrate perspective and emotional intelligence, and navigate complex interpersonal dynamics [1,4,5].

Despite their widespread adoption, the development of high-quality MMI stations remains challenging. The intense competition for limited places requires a large and continually refreshed pool of MMI stations to maintain test security and ensure fairness for all applicants. Developing and renewing this pool through traditional methods is resource intensive and places a considerable burden on faculty, while rushed or inconsistent development across multiple contributors can compromise station quality and reliability [6]. Artificial intelligence (AI) offers a potential solution to these challenges by streamlining station development and supporting the creation of a more reliable and scalable pool of assessment tools [7,8]. Prior work has shown that AI can generate assessment items or scenarios with acceptable quality or psychometric properties [7,9]. However, to date, no published studies have systematically evaluated the effectiveness of AI-generated MMI stations in real-world admissions settings.

In this study, we performed a comprehensive reliability analysis on 17 new MMI stations generated with the assistance of AI (AI-generated stations) and 39 existing MMI stations developed from non-AI sources used in the selection process for the 2025 intake of domestic students into the Direct Entry and Graduate Entry Medicine Program at Monash University. Using Cronbach α to assess internal consistency and SD as well as range of scores to evaluate discriminative capability, our analysis aimed to explore the reliability and overall quality of individual stations, uncover differences in performance between AI-generated and traditional stations, and identify stations that warrant modification, replacement, or serve as models for future development. These insights can be used to guide evidence-based improvements in MMI design and the integration of AI in medical admissions.

## Methods

### Study Design

This study analyzed 56 MMI stations used in the selection process for the 2025 admissions cycle of domestic applicants into the Direct Entry and Graduate Entry Medicine Program at Monash University. The 56 stations were divided into two categories: (1) AI-generated stations (n=17, 30.4%), and (2) existing or traditional stations (n=39, 69.6%). Each station was mapped to one of 7 MMI domains: advocacy, collaboration, critical thinking, empathy, ethical reasoning, motivation, and resilience. These domains map to desirable qualities of successful applicants and are used to guide the focus of the stations. They are not statistically determined discrete constructs within the MMI.

Traditional MMI stations were existing scenarios previously used in admissions and developed through established faculty-led processes. AI-generated MMI stations were developed using Claude 3.5 Sonnet (Anthropic), a proprietary, closed-source AI platform. Claude was selected for this study primarily because of its data privacy policy at the time of the project, which specified that user inputs were not retained or used for model training. Given that examination materials were included in prompts, this provided additional assurance regarding the protection of assessment content and institutional data.

The AI platform was provided with the following user-generated prompt:

*Please generate an interview based on the provided document. The interviews are for high-school students who are applying for medical school and test the student’s capacity for critical thinking. The interview structure should have a single paragraph for the applicant scenario, a single paragraph describing key components of the scenario for interviewers and 5 questions, with dot points on what an excellent response should look like*.

We designed this prompt to elicit realistic, contextually appropriate, and assessment-relevant content for medical school admissions, and guided the AI in producing full scenarios, associated interview questions, and example candidate responses.

To ensure content validity, relevance, and alignment with assessment objectives, all AI-generated scenarios underwent a structured quality assurance process. A working group of academic staff involved in admissions across both the Australian and Malaysian campuses reviewed the AI-generated stations as part of this process. Each scenario was independently reviewed by 2 faculty members from this working group with expertise in medical education and admissions. Reviewers evaluated alignment with the intended competency domain, clarity of the scenario, and potential sources of bias or ambiguity. Feedback from these reviews was incorporated through iterative refinement of prompts and station content. Final approval was contingent upon a consensus decision made jointly by 2 senior academic leads, providing an additional layer of oversight and quality control. While this process mitigates some risks inherent to AI-generated content, it does not eliminate potential biases associated with proprietary large language models, which lack transparency regarding training data and internal parameters [10]. Example traditional and AI-generated MMI stations from the collaboration domain, including scenario prompts and structured questions, are provided in Multimedia Appendix 1.

### Data Collection

Data were obtained from MMIs conducted between September 2024 and January 2025 on 824 domestic applicants, yielding a total of 4897 applicant-station interactions. Applicants were randomly assigned to a subset of the 56 available stations (Multimedia Appendix 2), which were distributed across multiple interview days. Each MMI station was scored on a 20-point scale, consisting of 5 questions with each question scored from 0 to 4 points.

A total of 252 interviewers participated in the interview process across both direct and graduate entry admissions. All interviewers completed standardized training prior to the MMI cycle. Interviewers were drawn from a range of backgrounds including health professionals, academics, community representatives, and occasionally senior students or recent graduates. Interviewers were recruited through an expression-of-interest process and screened for eligibility and potential conflicts of interest. Training included orientation to the MMI process, a scoring framework, and interviewer responsibilities through preparatory materials and live workshops. On each interview day, interviewers also participated in station-specific calibration sessions with other assessors assigned to the same station to promote consistent interpretation of scoring criteria.

Each candidate was assessed by a single trained interviewer at each MMI station. Multiple interviewers were assigned to each station across interview sessions, with the number of interviewers per station ranging from 2 to 17 depending on candidate volume per station. As each performance was rated by a single interviewer, interinterviewer reliability and interviewer variance were not examined in this analysis. To mitigate the risk of content dissemination between applicants, different stations were scheduled on different days.

### Reliability Analysis

To assess the reliability of individual MMI stations, we calculated Cronbach α for each station using applicant responses from the 2025 admissions cycle. Cronbach α ranges from 0 to 1 and is a measure of internal consistency; it reflects the extent to which items within an assessment measure the same underlying construct or set of related competencies [11-13]. In the context of a 5-item MMI station, higher α values indicate greater coherence among the scored questions, whereas lower values may suggest item misalignment, scoring inconsistency, or other sources of measurement error. Because α was calculated within each station in this study, it reflects the reliability of that station’s scoring rubric rather than the reliability of the overall MMI decision.

The categorization of Cronbach α scores for evaluating reliability remains a subject of debate, with many experts agreeing that thresholds should be determined by the purpose and stakes of the assessment. As Downing [13] noted, high-stakes assessments, such as licensure or certification examinations in medicine (which carry significant consequences for both candidates and the public), are generally expected to demonstrate reliability coefficients of 0.90 or above [13]. For assessments of moderate stakes, such as major summative examinations in medical school, a minimum reliability threshold of 0.80 to 0.89 is typically considered acceptable [12,13]. Lower-stakes assessments, including formative evaluations or internally developed tests used in teaching, may warrant reliability in the range of 0.70 to 0.79 [12,13]. Medical school admissions processes would generally be considered moderate- to high-stakes assessments, given their significant impact on applicants’ future careers and the responsibility of selecting candidates suited for a profession with substantial societal consequences. However, station-level reliability estimates in MMI formats must also be interpreted in light of the short rating scales used to score each station (5 items) and the multidimensional nature of competencies assessed within individual stations.

In line with this framework, we classified stations in this study into 3 conservative reliability groups as outlined in Table 1. These categories were used to support descriptive comparison and structured quality assurance rather than as binary standards of station usability. In this context, both optimal and acceptable meet expected reliability, while review indicates a station requires further examination.

**Table 1.** Reliability classification of multiple mini interview stations based on Cronbach α scores.

Reliability category^a^	Cronbach α
Optimal	≥0.85
Acceptable	0.75‐0.84
Review	<0.75

a Reliability categories were used for descriptive comparison and structured quality assurance review and do not represent binary standards of station usability.

### Discrimination Ability Analysis

In addition to reliability, we also evaluated station quality through discrimination capability, which was measured using the mean score, SD, and observed score range for each station. Discrimination refers to a station’s ability to distinguish or differentiate between candidates of varying competency levels across the scoring spectrum. Stations with adequate discrimination capability demonstrate higher SDs and broader score ranges, indicating they can effectively separate high-performing candidates from those with lower performance. Conversely, stations with poor discrimination capability exhibit low SDs or restricted score ranges, often due to ceiling effects (where most candidates score near the maximum) or floor effects (where most candidates score near the minimum). Such restricted score distributions limit a station’s utility for selection decisions, as they fail to provide meaningful discrimination between candidates regardless of their actual competency levels. While reliability ensures measurement consistency, discrimination capability determines whether that consistent measurement provides useful information for distinguishing candidate performance.

Reliability and discrimination metrics were analyzed as distinct psychometric properties but are reported together where appropriate to support integrated evaluation of station quality.

### Statistical Analysis

All statistical analyses were performed using R (version 4.5.1; R Foundation for Statistical Computing), primarily using the psych package for reliability analysis (Revelle, 2025; version 2.5.6) [14]. Figures were generated using GraphPad Prism (version 10.4.1; Dotmatics).

### Ethical Considerations

This study was conducted within the context of the standard medical school admissions process and aligned with routine quality assurance and program evaluation activities. Ethics approval was obtained from the Monash University Human Research Ethics Committee (49569). All applicant data were fully deidentified prior to analysis to protect participant confidentiality. As the study involved secondary use of deidentified administrative data, no additional consent from applicants was required.

## Results

### Overview

An overview of the internal consistency reliability assessed using Cronbach α and average scores, including the SD and range, for all 56 MMI stations is presented in Table 2. The following subsections outline key patterns and comparisons observed across station types and domains.

**Table 2.** Applicant scores and Cronbach α reliability rating for each of the 56 multiple mini interview stations used in the 2025 intake of domestic students at Monash University^a^.

Station code	Station type	Score, mean (SD)	Range	Cronbach α	Category
ADV_1	Existing	16.1 (3.0)	9-20	0.83	Acceptable
ADV_7	Existing	14.8 (3.2)	9-20	0.82	Acceptable
ADV_11	Existing	15.7 (2.8)	10-20	0.78	Acceptable
ADV_12	Existing	15.8 (2.8)	9-20	0.77	Acceptable
ADV_13^b^	Existing	14.2 (3.2)	6-20	0.85	Optimal
ADV_15	Existing	14.7 (2.9)	7-20	0.79	Acceptable
ADV_17	Existing	15.8 (3.3)	5-20	0.88	Optimal
ADV_18	Existing	12.6 (4.1)	6-20	0.89	Optimal
ADV_19	AI^c^ generated	11.2 (4.0)	0-19	0.85	Optimal
ADV_20	AI generated	14.5 (3.2)	7-20	0.78	Acceptable
ADV_21^b^	AI generated	17.3 (2.3)	14-20	0.75	Acceptable
COLL_1	Existing	15.8 (2.9)	9-20	0.83	Acceptable
COLL_2	Existing	15.6 (3.0)	8-20	0.76	Acceptable
COLL_3^b^	Existing	16.0 (3.1)	8-20	0.84	Acceptable
COLL_4^b^	Existing	15.6 (3.2)	8-20	0.82	Acceptable
COLL_9	Existing	16.0 (2.7)	10-20	0.77	Acceptable
COLL_10	Existing	16.0 (3.3)	0-20	0.87	Optimal
COLL_11	Existing	15.6 (3.2)	4-20	0.83	Acceptable
COLL_14^b^	Existing	17.3 (1.6)	15-20	0.39	Review
COLL_16	AI generated	14.7 (2.6)	8-20	0.76	Acceptable
CT_8.1	Existing	16.0 (3.2)	9-20	0.84	Acceptable
CT_9^b^	Existing	14.1 (3.9)	5-19	0.85	Optimal
CT_10	Existing	14.0 (3.5)	3-20	0.83	Acceptable
CT_13	Existing	14.9 (3.7)	4-20	0.88	Optimal
CT_16	Existing	15.1 (3.5)	7-20	0.88	Optimal
CT_27	AI generated	14.4 (3.4)	8-20	0.85	Optimal
CT_28	AI generated	14.2 (3.7)	1-20	0.88	Optimal
CT_29	AI generated	12.4 (3.4)	0-19	0.84	Acceptable
EMP_3^b^	Existing	15.3 (3.0)	8-20	0.77	Acceptable
EMP_6	Existing	14.2 (4.5)	3-20	0.93	Optimal
EMP_10	Existing	15.0 (3.4)	6-20	0.80	Acceptable
EMP_14	Existing	12.3 (4.0)	4-20	0.87	Optimal
EMP_15	Existing	16.0 (2.5)	9-20	0.73	Review
EMP_17	AI generated	13.8 (3.9)	2-20	0.90	Optimal
EMP_18	AI generated	15.7 (2.9)	6-20	0.82	Acceptable
EMP_19^b^	AI generated	13.2 (3.1)	6-20	0.82	Acceptable
ER_2	Existing	15.4 (3.2)	8-20	0.86	Optimal
ER_3	Existing	15.7 (2.8)	7-20	0.77	Acceptable
ER_6.1	Existing	14.2 (4.0)	5-20	0.88	Optimal
ER_16^b^	Existing	14.7 (3.6)	7-20	0.81	Acceptable
ER_24^b^	Existing	15.4 (2.6)	9-18	0.74	Review
ER_26	AI generated	15.9 (2.7)	8-20	0.69	Review
ER_27	AI generated	16.7 (2.5)	10-20	0.76	Acceptable
ER_28^b^	AI generated	13.9 (3.5)	6-20	0.86	Optimal
ER_29^b^	AI generated	14.4 (3.5)	7-19	0.86	Optimal
ER_30	AI generated	14.4 (3.5)	6-20	0.89	Optimal
MOT_2	Existing	14.8 (2.5)	6-19	0.66	Review
MOT_3	Existing	16.3 (3.5)	6-20	0.88	Optimal
MOT_4	Existing	15.5 (3.2)	5-20	0.84	Acceptable
MOT_6	Existing	17.0 (2.4)	11-20	0.76	Acceptable
MOT_9	Existing	15.2 (3.5)	6-20	0.87	Optimal
MOT_11^b^	Existing	14.6 (2.7)	7-20	0.69	Review
MOT_12^b^	AI generated	17.6 (2.1)	13-20	0.79	Acceptable
RES_1	Existing	15.4 (2.9)	9-20	0.84	Acceptable
RES_5	Existing	16.2 (2.9)	7-20	0.81	Acceptable
RES_6	AI generated	16.0 (3.0)	820	0.81	Acceptable

a Multiple mini interview stations were categorized into the following reliability categories, as outlined in Table 1: optimal (α≥0.85), acceptable (α=0.75-0.84), and review (α<0.75).

b Indicates stations with fewer than 50 candidate responses. Cronbach α estimates are generally less stable in small samples, and results for these stations should therefore be interpreted with caution.

c AI: artificial intelligence.

### Overall Reliability of AI-Generated and Traditional Stations

**Figure 1.**
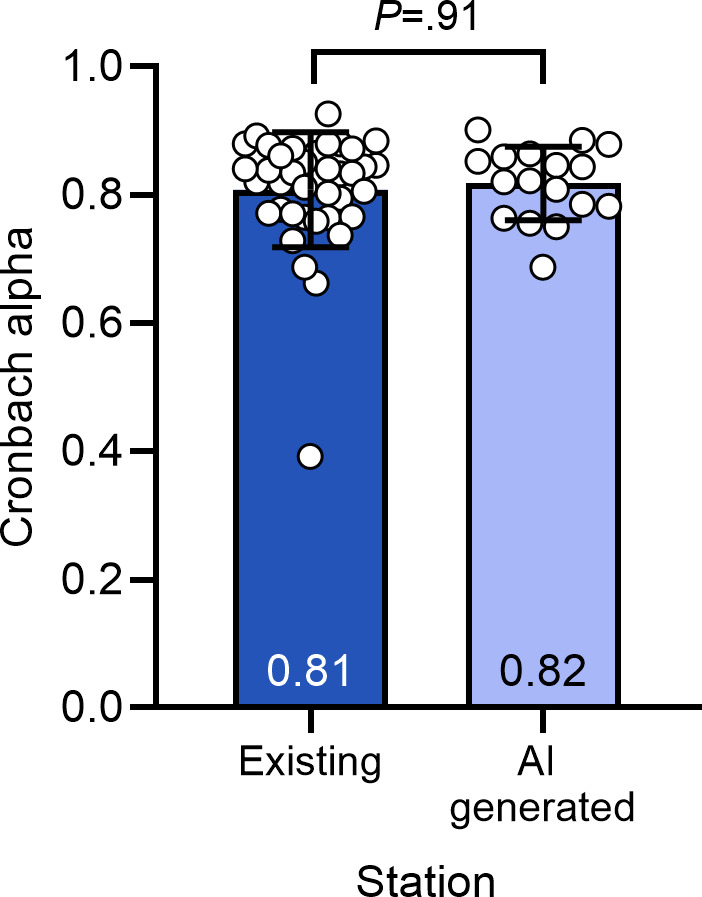
Comparison of internal consistency reliability (Cronbach α) between artificial intelligence (AI)–generated and traditionally developed multiple mini interview stations.

We conducted a Mann-Whitney *U* test to compare the internal consistency reliability of AI-generated and traditionally developed MMI stations. On average, the AI-generated stations demonstrated slightly higher Cronbach α values (mean α=0.82, SD 0.06) than the existing stations (mean α=0.81, SD 0.09). However, this difference was neither statistically significant (*U*=325; *z*=–0.116; *r*=−0.015; *P*=.91) nor was the difference large enough to be practically relevant (Figure 1). These findings suggest that while AI-generated stations may show marginally higher reliability, the overall level of internal consistency is comparable between the 2 station types.

In Figure 1, the mean Cronbach α values for AI-generated (17/56, 30%; mean α=0.82, SD 0.06) and traditional (39/56, 70%; mean α=0.81, SD 0.09) stations used in the 2025 Monash University medical school domestic admissions cycle. Bars represent mean (SD). *P* value was determined using a Mann-Whitney *U* test (*U*=325; *z*=−0.116; *r*=−0.015; *P*=.91).

**Figure 2.**
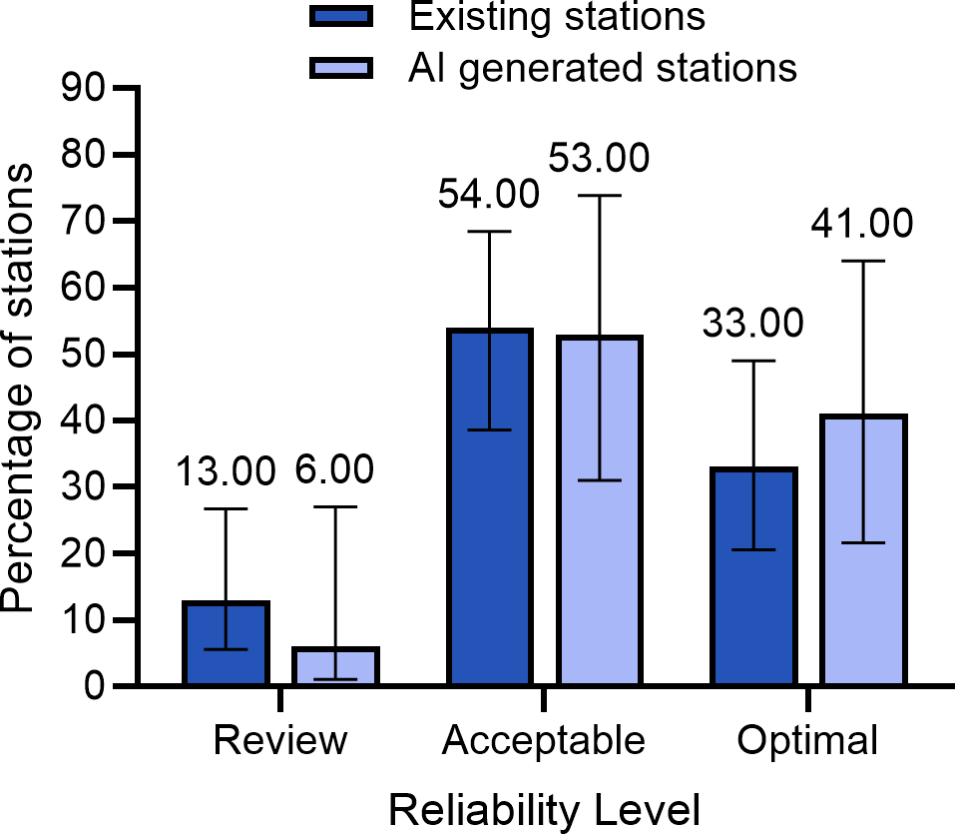
Distribution of reliability categories for artificial intelligence (AI)–generated and existing multiple mini interview stations.

When categorizing stations into review, acceptable, or optimal reliability groups (Table 1), AI-generated stations showed a slightly greater proportion achieving optimal reliability, with 41.2% (7/17; 95% CI 22%‐64%) of AI-generated stations meeting this threshold compared with 33.3% (13/39; 95% CI 21%‐49%) of the traditionally developed stations (Figure 2). Additionally, the proportion of AI-generated stations in the review category was 5.9% (1/17; 95% CI 1%‐27%) and was lower compared with traditionally developed stations of which 12.8% (5/39; 95% CI 6%‐27%) were in the review category (Figure 2). Most existing and AI-generated stations exhibited acceptable reliability, comprising 53.8% (21/39; 95% CI 39%‐68%) of traditionally developed stations and 52.9% (9/17; 95% CI 31%‐74%) of AI-generated stations (Figure 2). However, CIs were wide and substantially overlapping across categories, indicating that these proportional differences should be interpreted with caution.

To assess the influence of small sample sizes on station categorization, we conducted a sensitivity analysis excluding stations with fewer than 50 participants. When restricted to stations with n≥50, there remained no significant difference in internal consistency between AI-generated and traditionally developed stations (*P*=.89). The distribution of reliability categories was similar between groups, with 41% (7/17) of AI-generated and 37% (33/39) of traditionally developed stations classified as optimal, 53% (9/17) and 54% (21/39) classified as acceptable, and 6% (1/17) and 13% (5/39) classified as review, respectively. These findings indicate that category-based differences observed in the full dataset are sensitive to the inclusion of small-N stations. All subsequent analyses were conducted using the full dataset unless otherwise specified.

In Figure 2, the proportion of stations classified as review, acceptable, and optimal based on Cronbach α is shown for AI-generated (17/56, 30%) and existing or traditionally developed stations (39/56, 70%). Percentages were calculated within each station type, relative to the total number of stations in each category. Error bars represent 95% Wilson CIs for the proportion of stations in each reliability category.

To address potential confounding factors and ensure that apparent differences were not due to unequal distribution of AI-generated stations across MMI domains, we conducted a linear regression analysis. This analysis evaluated the association between station type and Cronbach α while controlling for MMI domain, essentially asking whether AI-generated stations would still show different reliability if both station types were equally represented across all domains. Although Cronbach α is bounded between 0 and 1, observed values in this dataset were not concentrated near the boundaries (range 0.39‐0.93). Linear regression was therefore considered appropriate for estimating adjusted mean differences in α. The analysis showed that station type was not a significant predictor of reliability (β=−0.003; *P*=.90), indicating that the slight advantage observed for AI-generated stations was not attributable to systematic differences in station development approaches. A beta regression model was also fitted as a sensitivity analysis; this yielded the same substantive inference (*P*=.77).

The regression analysis also examined whether some domains were inherently more challenging for developing reliable stations, which could have explained apparent station type differences if AI-generated stations were concentrated in naturally high-reliability domains. However, no significant differences in reliability were observed between domains (all *P*>.10), indicating that critical thinking, empathy, advocacy, and other domains showed similar reliability levels regardless of station type.

Finally, we performed an interaction model to test whether AI-generated stations might have domain-specific strengths or weaknesses compared with traditional stations; for example, whether AI might excel at generating critical thinking stations but struggle with empathy scenarios. This analysis found no significant interactions (all *P*>.48), demonstrating that the relationship between station type and reliability remained consistent across all MMI domains.

Collectively, these analyses demonstrate that while AI-generated stations showed slightly higher descriptive reliability metrics across multiple measures, these differences were not statistically significant. The findings suggest that AI-generated station development produces reliability outcomes equivalent to traditional faculty-led development methods, with performance remaining consistent across different MMI domains.

### Station Quality Analysis: Reliability and Discrimination Ability

Analysis of individual station performance revealed notable variation in both reliability and discrimination characteristics across AI-generated and traditionally developed stations. Several stations demonstrated exceptional reliability, with Cronbach α values exceeding 0.90 (Table 2). Notably, EMP_17 (α=0.90), an AI-generated station, and EMP_6 (α=0.93), a traditionally developed station, exhibited the highest internal consistency. Furthermore, both EMP_17 (mean 13.8, SD 3.9; range 2‐20) and EMP_6 (mean 14.2, SD 4.5; range 3‐20) stations demonstrated excellent discrimination capabilities. Other stations showing this desirable combination of high reliability and strong discrimination included ADV_18 (α=0.8925; mean 12.6, SD 4.1) and ER_6.1 (α=0.88; mean 14.2, SD 4.0). These stations may serve as exemplars for future MMI station development. Their structure was characterized by clear and focused prompts, as well as well-defined evaluation criteria, contributing to consistent assessment across different evaluators. The high reliability and discriminatory ability of these stations suggested they were effective in eliciting and measuring the targeted competencies in a robust and reproducible manner.

Conversely, some stations with acceptable reliability showed limited discrimination capability. For example, ADV_21 (α=.75; mean score 17.3, SD 2.3) and MOT_12 (α=.79; mean score 17.6, SD 2.1) exhibited ceiling effects, with most candidates scoring in the upper range of the scale, limiting their ability to distinguish between high-performing candidates (Table 2). While these stations demonstrated reasonable consistency, their restricted score distributions suggested they may be too easy or lack sufficient challenge to effectively discriminate between candidates of differing ability levels.

Notably, the traditionally developed station COLL_14 demonstrated poor performance across both metrics, with critically low reliability at a Cronbach α of 0.3925, well below the acceptable threshold, and poor discrimination (mean 17.3, SD 1.6; range 15‐20), with scores clustering near the maximum possible score (Table 2). Although this station had a limited sample size (18/824, 2%), the combination of poor reliability and severely restricted score range suggested fundamental issues with station design or implementation that warranted complete revision or replacement.

In comparison, the lowest-performing AI-generated station, ER_26, showed suboptimal reliability with a Cronbach α of 0.6876, but maintained reasonable discrimination (mean 15.8, SD 2.7; range 8‐20), suggesting that while this station requires refinement for internal consistency, it retains adequate discriminative function across the scoring range.

It should be noted that stations ADV_21, MOT_12, and COLL_14 had fewer than 50 candidate responses (Multimedia Appendix 2), which can result in less stable reliability estimates. Thus, station-level Cronbach α values for these stations should be interpreted with caution. Nevertheless, taken together, these results demonstrate that both AI-generated and traditional development methods are capable of producing stations across the full spectrum of psychometric quality, from exemplary stations that excel in both reliability and discrimination to problematic stations requiring revision or replacement. The findings suggest that station effectiveness depends primarily on individual design characteristics and implementation rather than the development methodology used.

## Discussion

### Principal Findings

This study compared the psychometric performance of AI-generated and traditionally developed MMI stations used in a high-stakes medical school admissions process. AI-generated stations demonstrated internal consistency reliability comparable to traditional stations, with no statistically significant differences in Cronbach α. A greater proportion of AI-generated stations were classified as optimal for internal consistency, while fewer fell within the review category. Both station types showed variable discrimination ability, indicating that overall station quality depended on individual design characteristics rather than development method. Taken together, these findings support psychometric equivalence between AI-generated and traditionally developed MMI stations rather than evidence of superiority.

### Implications for the Use of AI in Medical School Admissions

AI applications in medical education have shown promising results across various domains, including automated scoring of clinical examinations, natural language processing for medical curriculum development, and generation of multiple-choice questions for medical licensing examinations [15,16]. Similarly, AI has been increasingly integrated into admissions processes across professional programs, with applications ranging from automated essay scoring in standardized tests to predictive modeling of student success [17,18]. This study extends this emerging body of work by demonstrating that AI can produce psychometrically sound assessment content for high-stakes medical school selection processes. Unlike much of the existing literature, which focuses on AI for prediction or automated scoring of applicant performance, this study examines the use of AI for content generation, specifically the development of MMI stations themselves.

Our results showed that AI-generated stations demonstrated comparable internal consistency to traditionally developed stations (mean α=0.82, SD 0.06 vs mean α=0.81, SD 0.09; *P*=.91). Of note, a greater proportion of AI-generated stations were classified as optimal (α≥0.85), while fewer were classified in the review category (α<0.75) compared with traditional stations. However, this result is merely descriptive and should be interpreted with caution. The CIs around these proportions were wide and substantially overlapping, reflecting the small number of stations and indicating that the apparent differences are not statistically or practically robust. Nevertheless, these findings highlight the potential value of incorporating AI into admissions processes and provide a basis for examining how such tools might be further optimized and integrated into future selection frameworks.

Our findings align with broader research on AI-generated assessment content, which has consistently shown that the quality of AI-produced items depends more on human input in aspects including prompt engineering, content validation processes, and postgeneration refinement than on the AI system itself [19,20]. Studies comparing AI-generated and human-developed test items in educational contexts have similarly found equivalent psychometric properties when appropriate quality assurance measures are implemented [21,22]. This suggests that the observed variability in both AI-generated and traditional MMI stations reflects fundamental challenges in assessment development rather than limitations specific to either approach.

Analysis of discrimination capability revealed that both AI-generated and traditionally developed stations demonstrated variable performance in their ability to distinguish between candidates of different competency levels. Some stations from each development method exhibited excellent combinations of high reliability and strong discrimination, including AI-generated station EMP_17 and traditionally developed station EMP_6. Conversely, other stations from both approaches showed acceptable reliability but limited discrimination due to ceiling effects, where most candidates scored in the upper range of the scale. This variability suggests that station effectiveness depended primarily on individual design characteristics and implementation quality rather than the development methodology used.

Taken together, these findings underscore that while the AI-generated MMI development process shows great promise, it should be viewed as a complementary tool within a broader, evidence-based framework for admissions assessment rather than a replacement for expert oversight and continual quality assurance. Our observation that both development methods can produce stations across the full spectrum of psychometric quality emphasizes that ongoing review, calibration, and refinement remain essential for ensuring that all stations, irrespective of their origin, meet the rigorous standards necessary for fair and effective candidate evaluation.

A notable limitation of this analysis was the small sample size for certain stations (n<50), particularly for some of the new AI-generated stations. Smaller sample sizes can result in less stable reliability estimates, necessitating cautious interpretation of individual station-level Cronbach α values when analyzing the performance of specific stations. Although sensitivity analyses excluding small sample size (n<50) stations attenuated category-based differences, future reliability assessments should aim for minimum sample sizes of n≥50 per station to ensure more robust and stable estimates.

Nevertheless, the implications of these findings are particularly significant when considered in the context of the overall MMI station development process, which has traditionally been time-consuming, resource intensive, and reliant on substantial faculty input. Crafting high-quality stations requires not only content expertise but also careful attention to fairness, clarity, and alignment with assessment objectives; factors that can introduce variability and potential bias. The fact that AI-generated stations performed comparably to, and in some cases more reliably and with better discrimination ability than, traditionally developed stations suggests that AI may offer a scalable and efficient alternative for generating high-quality assessment scenarios. By streamlining the development process and reducing reliance on individual judgment, AI has the potential to support both the efficiency and equity of MMI design, while preserving the psychometric robustness of the assessment. Future research should formally evaluate whether AI-generated stations are truly noninferior to traditional stations regarding reliability, validity, and fairness, ideally through prospective, multi-institutional studies with larger sample sizes.

### Recommendations for Using AI in Future MMI-Based Admissions Processes

In this study, we have identified several actionable strategies to use AI to achieve quality and consistency in MMI station development. Drawing on the observed reliability data from both AI-generated and traditionally developed stations, the following recommendations are intended to guide future improvements in MMI design and implementation.

The continued use of AI-generated station development is supported by the data. AI-generated stations demonstrated excellent internal consistency and discrimination capability, indicating that this approach can produce high-quality assessment tools. Sustaining and expanding the use of AI in MMI design may enhance the efficiency and reliability of future station development while maintaining psychometric quality.

To preserve the psychometric integrity of the MMI system over time, a formal process for routine monitoring of both reliability and discrimination ability should be established. This process should involve the regular calculation of Cronbach α alongside assessment of score distributions and discrimination metrics, particularly for newly introduced or modified stations. Systematic monitoring will enable the timely identification of underperforming stations and facilitate data-informed decisions regarding their revision or replacement.

We further recommend standardization in station design. High-performing stations (eg, those with α>0.85 and high SDs) should be used as templates to guide the development of future scenarios. High-performing stations that demonstrate excellent reliability combined with strong discrimination capability (such as EMP_17 and EMP_6 in this study) should be used as templates to guide the development of future scenarios. Consistency in station structure, response criteria, and intended competencies is likely to improve fairness and reduce variability across MMI stations.

Stations that demonstrate below acceptable reliability (α<0.70) or limited discrimination capability should be prioritized for immediate review. This includes stations with low Cronbach α values (such as COLL_14) as well as those exhibiting ceiling effects that limit their discriminatory power (such as ADV_21 and MOT_12). Where appropriate, these stations should be revised or removed from the selection process. Additionally, for stations with limited response data (n<50), further data collection is necessary to obtain more stable reliability estimates and support evidence-based decision-making.

Finally, as the role of AI in MMI development expands, continual refinement with contemporary AI models will be essential. Improving the quality and contextual alignment of AI-generated content will require sustained collaboration between AI researchers and medical education professionals. This interdisciplinary approach will be critical to ensuring that AI tools not only support psychometric reliability but also align with the pedagogical and professional goals of medical school admissions.

### Conclusions

In this paper, we provide, to our knowledge, the first empirical evaluation of AI-generated MMI stations implemented in a real-world medical school admissions process, offering evidence supporting the use of AI-assisted methods in MMI station development. AI-generated stations demonstrated comparable reliability and discrimination capability relative to traditionally, entirely human-developed stations, with a higher proportion achieving optimal internal consistency and fewer falling below acceptable thresholds. Given the time- and resource-intensive nature of traditional station development, our findings suggest that AI may offer a scalable, efficient, and psychometrically sound alternative.

However, the observed variability in reliability and discrimination ability across both AI-generated and traditional stations underscores the necessity of ongoing quality assurance. Regular psychometric evaluation and iterative refinement remain essential to maintaining the validity and fairness of the MMI process. Looking ahead, the integration of AI into medical school admissions may extend beyond station generation to include broader applications such as predictive modeling of applicant success. Nonetheless, any expansion of AI use must be guided by careful consideration of ethical concerns, including transparency, algorithmic bias, and equity, to ensure that admissions systems remain fair and inclusive.

## Supplementary material

10.2196/86208 Multimedia Appendix 1 Example traditional and artificial intelligence–generated multiple mini interview stations from the collaboration domain, including scenario prompts and structured questions.

10.2196/86208 Multimedia Appendix 2 Detailed summary of multiple mini interview stations used in the 2025 Direct and Graduate Entry Medicine Program admissions cycle.

## Abbreviations

AI: artificial intelligence
MMI: multiple mini interview
