# Supplementary material for: AI-Generated Multiple Mini Interview (MMI) Stations for Medical School Admissions: Psychometric Evaluation
Source: JMIR Med Educ. 2026 Jun 11;12:e86208. doi: 10.2196/86208 (PMC13256173; doi:10.2196/86208)
Supplement: Multimedia Appendix 1 [file mededu-v12-e86208-s001.docx]

**COLL_9 – Kathy’s Fall**

**Domain:** Collaboration

**Station Type:** Traditional

**Applicant Scenario:**

Kathy is a star athlete on the relay team. Three days ago, she fell during practise, but assured her coach and teammates she was fine. Since then, her knee has become increasingly painful. The team is relying on her for next week’s championship. Kathy has trained long and hard for this championship race and is determined not to let anything prevent her from participating.

**Questions:**

1. What are Kathy’s options and what should she do now?
2. In your view does Kathy have an obligation to inform her coach of her injury? Why or why not?
3. What are some possible consequences of a decision to run despite the injury?
4. In life, how important is it to balance personal and team goals? Explain.
5. Describe a time when you have worked in a team and the outcome was not as positive as you had hoped?

**COLL_16 – Online Learning Platform**

**Domain:** Collaboration

**Station Type:** AI-Generated

**Applicant Scenario:**

Your school has decided to implement a new online learning platform to supplement in-class instruction and provide students with additional resources. As a member of the student technology committee, you have been selected to join a team of five students tasked with providing input on the platform’s features, testing the system, and helping to roll out the platform to the student body. However, you notice that some team members are struggling to adapt to the new technology and are resistant to change, leading to conflicts within the group.

**Questions:**

1. How would you approach team members who are resistant to change and struggling to adapt to the new online learning platform?
2. What strategies would you suggest to help team members overcome technological challenges?
3. How would you handle conflicts that arise within the team due to differing opinions on the platform’s features or implementation?
4. How can you foster a sense of collaboration and unity among team members, despite the challenges faced during the project?
5. What lessons about collaboration and adapting to change do you think this experience could teach the team?
