# Supplementary material for: AI-Generated Multiple Mini Interview (MMI) Stations for Medical School Admissions: Psychometric Evaluation
Source: JMIR Med Educ. 2026 Jun 11;12:e86208. doi: 10.2196/86208 (PMC13256173; doi:10.2196/86208)
Supplement: Multimedia Appendix 2 [file mededu-v12-e86208-s002.docx]

| **Table S1. Detailed summary of MMI stations used in the 2025 Direct and Graduate Entry Medicine Program admissions cycle.** | | | | |
| --- | --- | --- | --- | --- |
| Domain | Station Code | Station Type | Entry Program | Candidates |
| Advocacy | ADV_1 | Existing | Graduate | 95 |
|  | ADV_7 | Existing | Direct | 95 |
|  | ADV_11 | Existing | Graduate | 72 |
|  | ADV_12 | Existing | Graduate | 96 |
|  | ADV_13* | Existing | Graduate | 48 |
|  | ADV_15 | Existing | Direct | 96 |
|  | ADV_17 | Existing | Direct | 94 |
|  | ADV_18 | Existing | Direct | 50 |
|  | ADV_19 | AI-generated | Direct | 94 |
|  | ADV_20 | AI-generated | Direct | 67 |
|  | ADV_21* | AI-generated | Direct | 18 |
| Collaboration | COLL_1 | Existing | Direct | 95 |
|  | COLL_2 | Existing | Direct | 94 |
|  | COLL_3* | Existing | Graduate | 47 |
|  | COLL_4* | Existing | Direct | 48 |
|  | COLL_9 | Existing | Direct | 67 |
|  | COLL_10 | Existing | Direct & Graduate | 166 |
|  | COLL_11 | Existing | Direct & Graduate | 192 |
|  | COLL_14* | Existing | Direct | 18 |
|  | COLL_16 | AI-generated | Direct | 96 |
| Critical Thinking | CT_8.1 | Existing | Direct | 94 |
|  | CT_9* | Existing | Direct | 48 |
|  | CT_10 | Existing | Direct | 96 |
|  | CT_13 | Existing | Direct & Graduate | 117 |
|  | CT_16 | Existing | Direct & Graduate | 90 |
|  | CT_27 | AI-generated | Direct | 96 |
|  | CT_28 | AI-generated | Direct & Graduate | 190 |
|  | CT_29 | AI-generated | Direct | 94 |
| Empathy | EMP_3* | Existing | Direct | 18 |
|  | EMP_6 | Existing | Direct | 94 |
|  | EMP_10 | Existing | Direct & Graduate | 190 |
|  | EMP_14 | Existing | Direct | 96 |
|  | EMP_15 | Existing | Direct | 67 |
|  | EMP_17 | AI-generated | Direct & Graduate | 141 |
|  | EMP_18 | AI-generated | Direct & Graduate | 169 |
|  | EMP_19* | AI-generated | Direct | 48 |
| Ethical Reasoning | ER_2 | Existing | Direct | 94 |
|  | ER_3 | Existing | Direct | 96 |
|  | ER_6.1 | Existing | Direct | 95 |
|  | ER_16* | Existing | Graduate | 47 |
|  | ER_24* | Existing | Direct | 18 |
|  | ER_26 | AI-generated | Direct | 67 |
|  | ER_27 | AI-generated | Direct | 96 |
|  | ER_28* | AI-generated | Graduate | 47 |
|  | ER_29* | AI-generated | Direct | 48 |
|  | ER_30 | AI-generated | Direct & Graduate | 189 |
| Motivation | MOT_2 | Existing | Graduate | 72 |
|  | MOT_3 | Existing | Direct | 67 |
|  | MOT_4 | Existing | Graduate | 96 |
|  | MOT_6 | Existing | Direct | 96 |
|  | MOT_9 | Existing | Direct | 94 |
|  | MOT_11* | Existing | Direct | 48 |
|  | MOT_12* | AI-generated | Direct | 18 |
| Resilience | RES_1 | Existing | Direct | 96 |
|  | RES_5 | Existing | Direct | 96 |
|  | RES_6 | AI-generated | Direct | 95 |

Advocacy (ADV), Collaboration (COLL), Critical Thinking (CT), Empathy (EMP), Ethical Reasoning (ER), Motivation (MOT), Resilience (RES). * Indicates stations with fewer than 50 candidate responses.
